# Analecta

**Published:** 1842-04-02

**Authors:** 


### ANALECTA.

*On Croton Oil in Tic Douloureux.* By J. A. Easton, M., D., Professor of Materia Medica in Anderson's University, &c.—The observations of Janelli, of Sir Charles Bell, Dr. Newbigging, Mr. Cochran (of Edinburgh,) and others, on croton oil as a remedy in nervous diseases, induced me to try that medicine in the subjoined case which presented itself on the 10th inst. On that day I was requested to visit Mr. W. M., a gentleman, whose vocation as a commercial traveller necessarily exposes him to the full force of those sudden atmospheric changes which take place so frequently in our northern climate. Four days before this, Mr. M. had travelled for five hours, from five to ten, P. M., on the top of a coach during the prevalence of a piercing easterly wind. The day after this journey, he was seized with intense pain in the left side of the head; to relieve which, he applied, of his own accord, eight leeches to the affected part, and had recourse to Epsom salts and other purgatives. Deriving no benefit from his stock of *domestic therapeuticals*—which he had completely exhausted—my services were requested on the 10th, as already mentioned. Pain, represented as excruciating and darting, is experienced at stated periods in the left side of the scalp from forehead to vertex, while pressure on the trunk of the left supraorbital nerve augments his sufferings to an almost intolerable degree. The headach commences about five in the afternoon, and continues without intermission or abatement for fourteen hours, during which the patient is so distractingly agonised that he feels a strong desire to dash his head on the wall, or any other solid body that is within his reach. His friends state, that at this period he is slightly incoherent. With the termination of the dreaded fourteen hours, return tranquillity of mind and alleviation of pain. The bowels are regular, the pulse is 80, the skin cool, the tongue whitish; but it ought to be mentioned, that when these observations were noted, the headach, though severe, was comparatively tolerable. The following was ordered :—

℞ Croton oil, gtts. ij.
Compound extract of Colocynth, gr. xij.

Make into four pills, of which let one be taken every two hours.

On the next day (the 11th) the headach was greatly relieved, though by no means removed. The medicine, to use his own language, had produced "above forty stools," of a yellow colour and most offensive smell. The urine was greatly increased in quantity, and of a deep-red colour. Desirous to follow up the success which had been evidently obtained, I ordered the pills to be repeated; but the severe purgation which they had induced formed an obstacle to their administration, which neither argument nor entreaty could overcome. Under these circumstances the treatment at first adopted was abandoned, and the following was substituted:—

℞ Arsenical solution, gtts. viij. Three times a day.

12. Pain of head returned yesterday afternoon with nearly equal intensity, and Mr. M. has passed a sleepless night. Will not consent to take the pills which were prescribed on the 10th. Increase the dose of the solution of arsenic to ten drops; and let half a drachm of tincture of aconite be rubbed upon the painful part of the head, morning and evening.

13. No change. Headach as severe as formerly, and of the same duration.

14. Headach as intense as on the 10th. Patient, having an impression that death will soon terminate his sufferings, will now submit to anything in the way of treatment. Omit the solution of arsenic and tincture of aconite.

℞ Croton oil, gtts. ij.
Crumb of bread, q. s.

Make into four pills; one to be taken every three hours.

15. Pain of head did not return until five hours after the usual period, and, when it did commence, was less severe; alvine evacuations frequent, but neither so copious nor so offensively fœtid as formerly, and of natural colour; urine greatly increased in quantity. Continue the pills.

16. Had only four hours of pain which was moderate and tolerable; alvine evacuations abundant, but not profuse; no note of state or amount of urine. Continue the pills.

17. Has had no headach since last visit; slept well last night, and is refreshed, composed, and cheerful. Take one pill night and morning.

18. No return of headach; feels perfectly well, and is anxious to resume business. Pills produced no greater action upon the bowels than what an ordinary laxative might have been supposed to have induced.

24. Continues free from headach up to this date.

*Remarks.*—That the benefit in this case was owing to the croton oil is evident, I think, from the circumstance, that this medicine was the first thing to make an impression on the disease, which had resisted ordinary purgatives and the application of leeches to the head; and further, that when the oil was intermitted, the headach returned with its former intensity—yielding neither to arsenic—valuable so frequently in such cases—nor to that excellent anodyne, the aconitum napellus. Secondly. That the beneficial effects of the remedy were not owing to its action as a *mere purgative*, but to something specific in regard to the disease for which it was administered, appears likely from the circumstance, that this patient had previously had recourse to the more usual purgatives, had induced profuse catharsis, yet experienced no mitigation of suffering. Thirdly. In what this specialty of the croton tiglium consists, or through what media it developes itself, I am unable to say; but I cannnot help calling attention to the circumstance, that the urine was increased in quantity, and was evidently denser than usual, and that the alvine evacuations were of a "yellow colour and most offensive smell."

Now these are precisely the more important physiological actions of *colchicum autumnale*, so valuable an agent in articular rheumatism, to which, in my opinion, neuralgia bears a strong resemblance. The seat of this latter ailment is, I conceive, in the fibrous neurilema, while under the influence of that modified form of inflammation which is set up in the fibrous structures of the joints when they are attacked with rheumatism. Not only does identity of tissue support this view of the pathology of the disease, but similarity of symptoms also, particularly the characteristic tendency in both maladies to periodical exacerbations and to intervals of comparative repose. Dr. Lewins, of Leith, has demonstrated from chemical analysis, that by the exhibition of the meadow-saffron the specific gravity of the urine occasionally rises from 1.000 to as high as 1.037; and that the cause of this increased density is the augmentation of urea and of urate of ammonia, results which Dr. Lewins tells us have been verified by Professor Chelius, of Heidelberg. By the influence of colchicum also, as well as by that of croton tiglium, the alvine evacuations assume a bright yellow colour, the liver being stimulated apparently through the duodenum, in accordance with the physiological law, that when a membrane is irritated on which an excretory duct opens, the gland from which that duct proceeds is excited to unusual secerning activity. Can it be then that the croton tiglium is similar in its action to the colchicum autumnale, and that it does good in *tic douloureux* by inducing the same effects that colchicum does when it alleviates the sufferings of the gouty and the rheumatic—by eliminating urea and uric acid salts through the urine when these highly nitrogenous productions of the blood are in excess, and thereby the sources of constitutional irritation, and also by causing a supersecretion of bile? We know that colchicum is an invaluable remedy in fibrous or articular rheumatism; and if I am correct as to the pathology of neuralgia, and if croton oil is beneficial in that disease, is it unreasonable to suppose that two remedies which cure similar complaints should do so in a similar manner?

This case and these speculations have been published chiefly for the purpose of directing the attention of the profession to this subject, to the effects especially of croton oil on the kidney, and to the character of the urine under its influence; for I feel conscious that I did not in the above case examine that excretion so minutely, as to warrant me in trespassing any longer on the indulgence of the reader.—*London Lancet*, Jan. 29, 1842.

[We have used the croton oil as a purgative in congestions of the brain, marked by flushed face and attended with pain of a neuralgic character, but not in simple neuralgia. In the former class of cases, it is a most valuable remedy. In a patient to whom we lately gave it, the medicine relieved him after two doses, when bleeding and purgatives had failed. There is evidently something peculiar rather than specific in the action of the medicine. When first taken it produces a strong feeling of nausea, and purges some time afterwards; if it be frequently repeated, the nausea does not diminish at each dose, although the purgative power does. There is certainly much analogy between its action and that of colchicum.]

---

*Abscess in the Pelvis after Parturition.* By J. T. Jameson, Surgeon to the Rochdale Dispensary.—Mrs. N—— was admitted a patient of the dispensary under my care, November 3, 1841. She informs me that it is a month since her confinement, in which she was attended by a female, claiming the title of a midwife. The labour was speedy, and the placenta not being equally so in its exit, the midwife pulled it away with much violence, giving her acute pain, particularly in the left iliac region. There was a copious discharge of blood at the time, and a moderate quantity for a few days subsequently. The pain in the left groin, for on more particular examination such was found to be its seat, has been gradually increasing from the time of delivery, and, according to her description, is of a throbbing and occasionally stabbing character, shooting down to the anus, and across to the pubes. In the horizontal position the pain is not so violent except at night, when it is frequently very severe, and deprives her of sleep. When she assumes the erect position, the pain is very distressing, particularly on attempting to walk, which she accomplishes with the body bent forwards, and the left thigh drawn up towards the trunk. Bowels constipated, and when moved she experiences acute pain in the region of the anus; passes her urine with difficulty and much pain; countenance pale and haggard; pulse frequent and small; tongue dry and brown in the centre; thirst, loss of appetite, and emaciation. There is some degree of fulness to be detected in the left inguinal region; but no defined solid tumour can be felt. There is much pain experienced on pressure being made immediately above the centre of Poupart's ligament, and it is to this spot that she points as being the principal seat of her pain. A vaginal examination being permitted, no cervix uteri is to be felt, and the os tincæ is firmly closed. On the left side of the os uteri, a firm unyielding tumour is perceived, which projects but little into the vagina, and, so far as can be ascertained by the finger, appears to extend up towards the left groin. Pressure upon it produces acute pain; on examining per anum, a swelling, occupying chiefly the left side of the pelvic cavity, and apparently almost obliterating the calibre of the rectum, was discovered. Pressure upon the tumour causes here, also, severe pain. She was ordered to maintain the recumbent position and to take castor oil until the bowels were freely moved. Pil. sap. c. opii. grs. x. h.s.o.n.—Nov. 8. Found her sitting up, and much improved in appearance, having lost the haggard, anxious countenance above mentioned. The bowels have been well moved; she has much less pain, and is able to walk with the body nearly upright, and the whole of the left foot placed upon the ground, which she was unable to do before. Has slept much better the last two nights.—Whilst sitting up, on the evening of the 6th, she suddenly discharged about half a teacupful of pus per, vaginum, which was followed by immediate relief, and, on rising to walk, she found she was able to extend the thigh, and set down the foot much better than she could previously. She rapidly improved from this period, requiring merely a little castor oil occasionally; and on Tuesday, December 14, she came to the dispensary to be discharged.—She appears to walk without impediment, although she expresses herself as feeling weak on that side; with this exception, she seems quite restored.—*Provincial Medical & Sur. Journ. Jan.* 29, 1842.

